# Complete genome sequence of *Cereibacter sphaeroides* f. sp. denitrificans strain IL106

**DOI:** 10.1128/mra.00392-24

**Published:** 2024-07-17

**Authors:** Emma K. Stock, Kaziah J. Terrell, Amiera A. Rayyan, John A. Kyndt

**Affiliations:** 1 College of Science and Technology, Bellevue University, Bellevue, Nebraska, USA; California State University San Marcos, San Marcos, California, USA

**Keywords:** *Cereibacter*, *Rhodobacter*, denitrificans, complete genome, IL106

## Abstract

*Cereibacter sphaeroides* is a non-sulfur purple bacterium, one of the most versatile and thoroughly studied species of its kind, can grow on a variety of compounds photoheterotrophically, and is often used in bioremediation. We present the complete genome of *Cereibacter sphaeroides* f. sp. denitrificans underscoring its unique features.

## ANNOUNCEMENT

*Cereibacter* (*Rhodobacter*) *sphaeroides* f. sp. denitrificans was originally isolated from a polluted lagoon pond of Asahimatsu Frozen Bean-Curd Co. in Japan (1). The type strain of *Cba. sphaeroides*, strain 2.4.1, does not denitrify, but strain IL106 is well known to do so (1 – 4). In our own work, we found that IL106 does not make the usual SHP and PYP proteins found in the type strain (5, 6), but has homologs found in a distant *Rba. sphaeroides* relative, strain CZR27. The *Rhodobacter* genus taxonomy was recently restructured (7) and strain IL106 is now part of the newly described *Cereibacter* genus. Based on 16S rRNA, it is borderline a separate species from *Cba. johrii* (8). To help establish the phylogenetic position of IL106 and determine the genetic features underlying its unique capabilities, the complete genome sequence was determined.

Strain IL106 was isolated on YC-agar plates and selected on deep soft agar-tube cultures with nitrate as described in reference (1). We obtained the strain from Dr. Terry Meyer, who received it directly from the original authors. Genomic DNA was prepared directly from cells frozen at −80ºC in 50% glycerol, using the GeneJET DNA purification kit (ThermoScientific). The sequencing library was prepared using the Illumina DNA Library Prep kit and sequenced by an Illumina MiniSeq. Paired-end (2 × 150 bp) sequencing generated 9,584,560 reads and 1,158.6 Mbps. Quality control of the reads was performed using FastQC (v1.0.0) within BaseSpace (Illumina), using a k-mer size of 5 and contamination filtering. Oxford Nanopore DNA library prep was performed following the Ligation Sequencing Kit (SQK-LSK110) on a FLO-MIN106D flow cell with a MinION-Mk1B instrument (9). No DNA shearing was performed. Read QC and “superaccuracy basecalling” were performed using Guppy (v6.5.7) (10). This generated 106.3 Mbps sequencing data (55,075 reads), with an average read length of 2,323 bp. An assembly of the Nanopore sequencing was performed using Flye (v2.9.1) (11) within BV-BRC (12), resulting in eight contigs and an N50 of 3,193,481 bp. This initial assembly was of poor quality (fine consistency 65.2%) with too high CDS ratio (8,990). This long-read assembly was subsequently used as a reference sequence for the assembly of the Illumina reads by Minimap2 (13). 9,263,966 reads were aligned, resulting in an accumulative 183× coverage, and four contigs with an N50 of 3,207,152 bp. The coarse and fine consistency were improved to 98.2% and 94.3%, respectively. The final assembled genome was 98% complete according to CheckM (14) (Evaluation Group R200 *Rhodobacter sphaeroides* ATCC 17025). Each contig had overlapping repeat regions at the ends that confirmed circularity. The genome was annotated by the NCBI Prokaryotic Genome Annotation Pipeline (PGAP) (v6.6) (15). Default parameters were used unless otherwise specified.

*Cba. sphaeroides* IL106 is 4.79 Mb in size and the CG content is 69% (Table 1). The genome is composed of two chromosomes and two megaplasmids. Some of the other “*Rhodobacter”* species have shown to have two chromosomes, including the type strain 2.4.1 and more divergent species *Cba. azotoformans, Rba. sp. CZR27,* and *Cba. changlensis* (Table 1 and (16 – 19)).

**TABLE 1:** Overview of genome features of the complete genomes of *Cereibacter* species related to *Cereibacter sphaeroides* f. sp. denitrificans IL106^*a*^ ^,*b*^

Species	Size	% GC	Coverage	N50	CDS	tRNAs	ANI	Chrom 1	Chrom 2	Plasmids (Mb)	BioProject	Reference
*Cba. sphaeroides* IL106	4.8 Mb	69	183x	32,07,152	4944	54	-	3.2 Mb	1.33 Mb	0.13; 0.12	PRJNA483068	this study
*Cba. sphaeroides* HJ	4.6 Mb	69	25x	32,63,112	4510	55	97.7	3.3 Mb	1.19 MB	0.09	PRJNA523284	(20)
*Cba. sphaeroides* 2.4.1 [T]	4.6 Mb	69	/	31,88,609	4395	53	94.3	3.2 Mb	0.94 Mb	0.11; 0.11; 0.1; 0.1; 0.04	PRJNA40077	(16)
*Cba. azotoformans* ORIO	4.8 Mb	68.2	428x	30,35,272	4974	55	84.8	3.0 Mb	0.88 Mb	0.12; 0.14; 0.18; 0.3;0.14;0.04	PRJNA747035	(18)
*Rba.* sp. CZR27	4.4 Mb	68.5	100x	33,35,106	4335	54	82.2	3.3 Mb	0.86 Mb	0.19; 0.05	PRJNA401084	(19)

^*a*^ ANI percentage is based on bidirectional ANIb values to strain IL106, calculated using JSpecies (21).

^*b*^ “/” = not available.

**Fig 1:**
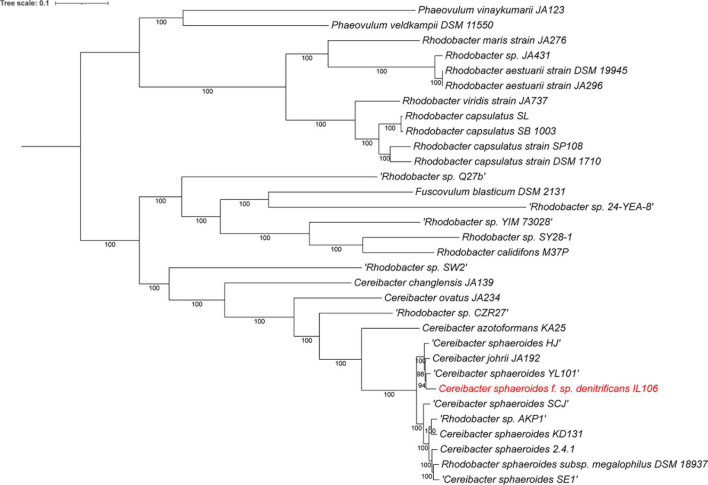
Phylogenetic tree of *Cereibacter sphaeroides* f. sp. denitrificans IL106 whole-genome comparison to its closest relatives. The phylogenetic tree was generated using the codon tree method within BV-BRC (11), which used PGFams as homology groups and analyzed 500 aligned proteins and coding DNA from single-copy genes using RAxML (**V8**) (22, 23). The support values for the phylogenetic tree are generated using 100 rounds of the “Rapid bootstrapping” option of RaxML. The branch length tree scale is defined as the mean number of substitutions per site, which is an average across both nucleotide and amino acid changes. iTOL was used for the tree visualization (24).

A JSpecies (21) ANI comparison of IL106 shows that *Cba. johrii, HJ*, and *YL101* are 97%–98% identical to one another, and 94% to 2.4.1 but only 82% to CZR27. Whole-genome phylogenetic analysis of the IL106 genome using RAxML (22, 23) showed *johrii, YL101,* and *HJ* as the closest relative (Fig. 1; (20)), and separated from the 2.4.1 and *azotoformans* branches.

## Data Availability

This genome project has been deposited at DDBJ/ENA/GenBank under BioProject PRJNA483068, with the following accession numbers: Chromosome1 CP147411; Plasmid2 CP147412; Chromosome2 CP147413; Plasmid1 CP147414. The raw sequencing reads have been submitted to SRA and the corresponding accession numbers for the Illumina data are SRR28616909, SRR28616908, and SRR29087253. The accession numbers for the Oxford Nanopore data are SRR28617170 and SRR28617169.
